# Supplementary material for: Association between oral microbiome and five types of respiratory infections: a two-sample Mendelian randomization study in east Asian population
Source: Front Microbiol. 2024 Apr 10;15:1392473. doi: 10.3389/fmicb.2024.1392473 (PMC11039966; doi:10.3389/fmicb.2024.1392473)
Supplement: Supplementary file 1 [file Data_Sheet_1.zip › Supplementary Materials/MR plots of saliva/Tonsillitis/s__Lancefieldella_sp000564995_mgs_2440/leave_one_out.pdf]

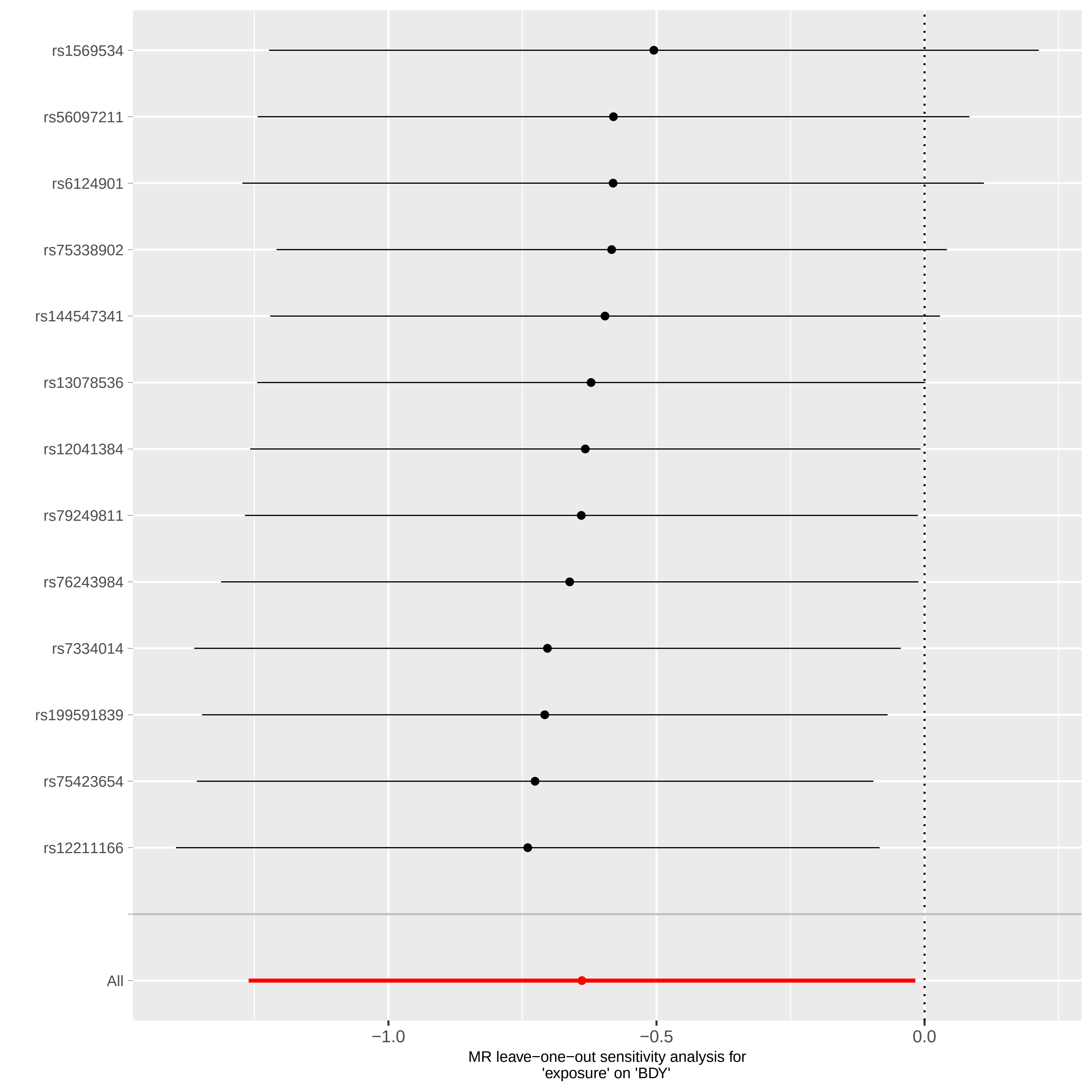
rs1569534
rs56097211
rs6124901
rs75338902
rs144547341
rs13078536
rs12041384
rs79249811
rs76243984
rs7334014
rs199591839
rs75423654
rs12211166
All
−1.0
−0.5
0.0
MR leave−one−out sensitivity analysis for
'exposure' on 'BDY'
